# Impact of Temporomandibular Joint Pain in Rheumatoid Arthritis

**DOI:** 10.1155/2013/597419

**Published:** 2013-12-09

**Authors:** Neveen Ahmed, Hamid Masoud Mustafa, Anca Irinel Catrina, Per Alstergren

**Affiliations:** ^1^Section for Orofacial Pain and Jaw Function, Department of Dental Medicine, Karolinska Institutet, P.O. Box 4064, 141 04 Huddinge, Sweden; ^2^Faculty of Medicine, Umm al-Qura University, Jeddah 21452, Saudi Arabia; ^3^Department of Rheumatology, Karolinska University Hospital, 171 76 Stockholm, Sweden; ^4^Specialized Pain Rehabilitation, Skåne University Hospital, 221 85 Lund, Sweden; ^5^Department of Orofacial Pain and Jaw Function, Faculty of Odontology, Malmö University, 205 06 Malmö, Sweden

## Abstract

To investigate the impact of temporomandibular joint (TMJ) pain on daily activities and quality of life in relation to systemic inflammatory activity in patients with rheumatoid arthritis (RA), thirty-three consecutive outpatients with RA were included. TMJ pain intensity at rest, on maximum mouth opening, and on chewing was assessed on a 0–10 numerical rating scale. TMJ palpatory tenderness, degree of anterior open bite, the impact of TMJ pain on daily activities and quality of life were also assessed. The systemic inflammatory activity was estimated by the disease activity score 28 (DAS28), blood levels of inflammatory markers and number of painful musculoskeletal regions. TMJ pain at rest, on maximum mouth opening, and on chewing as well as DAS28 was correlated with the impact of the TMJ pain on daily activities and quality of life. Partial correlations showed a significant interaction between TMJ pain on movement and DAS28 that explained the TMJ pain impact on daily activities and quality of life to a significant degree. This study indicates that both current TMJ pain intensity and systemic inflammatory activity play roles in the impact of TMJ pain on daily living and quality of life in RA.

## 1. Introduction

Pain is the major factor that reduces the quality of life for patients with rheumatoid arthritis (RA) [1]; for example, 66% of RA patients graded pain as the most important problem [2]. Pain is accordingly considered as the main reason for those patients to seek treatment [2].

The temporomandibular joint (TMJ) is often and early affected by RA [3]. For example Aliko and coworkers [4] found that 65% of RA patients have TMJ symptoms. The most common clinical finding is TMJ pain, especially on movement or loading. Involvement of the TMJ by RA may, besides pain, cause limitations of jaw function due to restriction of condylar translation. An anterior opening of the bite due to articular cartilage and bone tissue destruction may develop [5].

RA is a systemic disease with the major part of the pathology taking place in the synovial tissues. TMJ pain in RA has been found to be mediated and modulated by systemic as well as local mechanisms [6, 7]. However, the interaction between the systemic inflammatory activity and TMJ pain regarding its impact on daily activities and quality of life is not yet clearly understood.

*Aim.* The aim of this study was to investigate the impact of TMJ pain and inflammation on daily activities and quality of life in relation to the systemic inflammatory activity in patients with TMJ involvement of RA.

## 2. Materials and Methods

### 2.1. Patients

Thirty-three consecutive outpatients with RA at the Department of Rheumatology at Dr. Bakhsh Hospital, Jeddah, Saudi Arabia, were invited to participate in the study (Table 1). Patients were invited at first visits or follow-ups to the rheumatology clinic, without considering whether they had TMJ pain or not.

Inclusion criteria were diagnosis of RA according to the criteria of the American College of Rheumatology [8]. Exclusion criteria were age below 20 years, current malignancies, TMJ surgery, or trauma within one year and less than six months since an intra-articular corticosteroid injection in the TMJ.

Twenty-six of the patients agreed to participate in the full clinical examination and blood sampling. Seven patients declined to have blood samples taken but underwent the clinical examination. As a consequence, the number of observations in this study varies between 26 and 33.

The project was approved by the ethical committee of Ministry of Health, Jeddah, Saudi Arabia (H-02-J-002), which allowed this project to be conducted at Dr. Bakhsh Hospital. All subjects gave their informed consent before participation.

### 2.2. Clinical Examination

Each patient was clinically examined by one calibrated operator (Neveen Ahmed) that had no knowledge about the patient's general or rheumatological history before the examination. The clinical examination routine has been used in several previous studies [9, 10]. The examination performed in the present study comprised the following variables besides general information about age, sex, years since RA diagnosis and debut of TMJ symptoms, smoking habits, and medication.

DAS28 was obtained from the rheumatologist on the same day of the TMJ examination. DAS28 combines data from the swollen joint count, tender joint count, erythrocyte sedimentation rate, and the patient's self-evaluation of general health.

The patients were asked about present pain in nine joint regions besides the TMJ (neck, shoulders, elbows, hands, upper back, lower back, hips, knees, and feet) and the number of painful joint regions was recorded (score = 0–21).

A 0–10 numerical rating scale where 0 corresponded to “no pain” and 10 to “worst imaginable pain” was used to assess the TMJ pain intensity during the last week at rest, on maximum mouth opening, and on chewing for each TMJ. Maximal voluntary mouth opening capacity, laterotrusion to both sides, and protrusion were measured between 11 and 41 in mm. At the same time the numerical rating scale was used to assess pain intensity at maximum mouth opening, laterotrusion to both sides, and protrusion during the examination. The pain threshold was measured in mm as the maximum mouth opening without pain or without an increase of any ongoing pain. In the statistical analysis, the sum of the pain intensities on TMJ movements (maximal voluntary mouth opening, bilateral and contralateral laterotrusion, and protrusion) was calculated for each TMJ and added to a total score (0–40).

The patients were asked to estimate the impact of TMJ pain on their abilities to perform daily activities and on the quality of their life on a 0–10 NRS where 0 corresponded to “no impact of TMJ pain on daily activities/quality of life” and 10 corresponded to “maximum impact of the TMJ pain on daily activities/quality of life.”

A score (0–3) for tenderness to digital palpation of the TMJ with 5 N was adopted which involved evaluation of the lateral and posterior aspect of the joint on each side. For each site, one unit was scored if the patient reported tenderness upon palpation and two units were scored if the palpation in addition caused a palpebral reflex. The sum of these scores for each TMJ was calculated and used in the statistical analysis.

The degree of anterior open bite was used as a clinical marker of the degree of cartilage and bone destruction in the TMJ and it was assessed by recording of the occlusal contacts on each side upon hard biting in intercuspid position. The following scores were used on each side: 0 = occlusal contacts including the canine, 1 = no contacts anterior to the first premolar, 2 = no contacts anterior to the second premolar, 3 = no contacts anterior to the first molar, 4 = no contacts anterior to the second molar, and 5 = no occlusal contact. The sum of the scores on the right and left side was used in the analysis as an estimation of the degree of anterior open bite. None of the patients in our study was edentulous and the score thus ranged from 0 to 9.

### 2.3. Blood Sampling and Laboratory Procedures

Immediately after the clinical examination, venous blood (5 mL) was collected in uncoated tubes that were left in room temperature to coagulate for 1 hour and then centrifuged (10 min in 4°C at 1500 g). The supernatant (serum) was then stored (−80°C) until analysis. The erythrocyte sedimentation rate (ESR), C-reactive protein (CRP), anticitrullinated protein antibodies (ACPA), and rheumatoid factor (RF) concentrations as well as the thrombocyte particle count (TPC) were determined by the Saudi Laboratories at the Ministry of Health Research Center, Jeddah, Saudi Arabia.

ESR was analyzed with the Westergren method and levels below 20 mm/h for the female and below 13 mm/h for the male were considered normal. CRP was analyzed with the particle-enhanced turbidimetric test (Cobas Integra Analyzer, Roche, Mannheim, Germany). CRP levels below 5 mg/L were considered normal. ACPA was analyzed by a microparticle enzyme immunoassay for semiqualitative measurement of IgG class of autoantibodies specific for citrullinated proteins in human serum (Axsym, Abbott Laboratories, Abbott Park, IL, USA) and levels below 5 U/mL were considered as normal. RF was analyzed with the direct latex fixation test (Architect ci4100, Abbott Laboratories, Abbott Park, IL, USA) and levels below 14 IU/mL were considered normal. TPC was analyzed by The ADVIA 2120i System with Autoslide streamlined workflow (Siemens Medical Solutions USA, Inc., Malvern, PA, USA) and the reference range for normal value was 150–400 10^9^/L.

### 2.4. Statistics

Nonparametric statistics were used since the pain variables and the impact variables were considered to be measured on an ordinal scale. For descriptive statistics, median, 25th and 75th percentiles, and number of observations are reported. For relevant variables, the percentage of abnormal values is also reported. For analytical statistics, Mann-Whitney *U* test Spearman ranked correlation and partial correlation tests were used to calculate the significance of differences between groups and correlations between variables, respectively. For correlations between variables measured on an individual level with side-related variables the sum of the right and left side was used. A probability level of *P* < 0.05 was considered as significant.

## 3. Results

Table 1 shows the measures of systemic disease activity for the 33 patients with TMJ involvement of RA.  Table 2 shows the clinical findings in these patients.

Three (9%) of the patients reported no TMJ pain (rest, mouth opening, or chewing) at the visit.

Ten (32%) patients reported no TMJ pain at rest; six (19%) patients had no TMJ pain on maximum mouth opening, whereas eight (26%) patients had no TMJ pain on chewing.

Patients with TMJ pain at chewing had significantly higher DAS28 and number of painful regions than patients without TMJ pain (*P* = 0.036 and *P* = 0.022, resp.). Patients with pain on maximum mouth opening had significantly higher DAS28 (*P* = 0.037).

### 3.1. Impact of TMJ Pain on Daily Activities and Quality of Life

**Figure 1:**
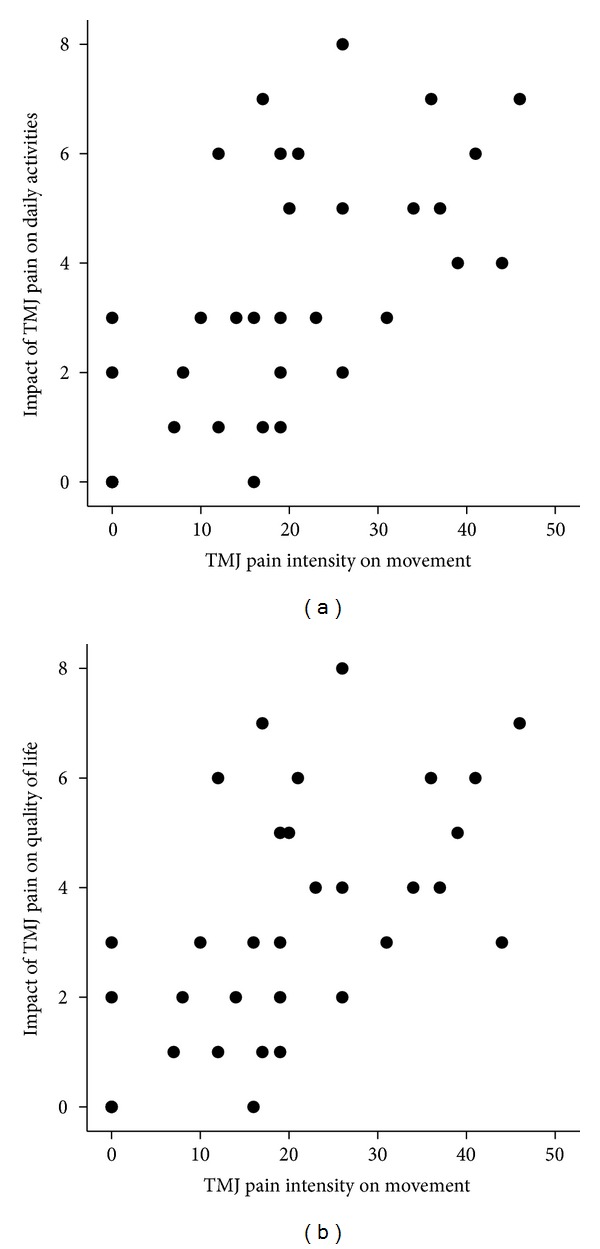
Scatter plots showing the relation between temporomandibular joint (TMJ) pain intensity on movement and the impact of TMJ pain on daily activities ((a): *r*_*s*_ = 0.72, *n* = 33, *P* < 0.001) and quality of life ((b): *r*_*s*_ = 0.73, *n* = 33, *P* < 0.001).

Four patients reported no restriction of activities of daily living by the TMJ pain. The TMJ pain variables, TMJ pain intensity at rest, on maximum mouth opening, and on chewing, had a strong impact on daily activities (*r*_*s*_ = 0.74, *n* = 33, *P* < 0.001, *r*_*s*_ = 0.72, *n* = 33, *P* < 0.001, and *r*_*s*_ = 0.72, *n* = 33, *P* < 0.001, resp.; Figure 1(a)) and quality of life (*r*_*s*_ = 0.70, *n* = 33, *P* < 0.001, *r*_*s*_ = 0.73, *n* = 33, *P* < 0.001, and *r*_*s*_ = 0.72, *n* = 33, *P* < 0.001, resp.; Figure 1(b)).

TMJ pain intensity on mandibular movements correlated with the impact on daily activities and quality of life (*r*_*s*_ = 0.64, *n* = 33, *P* < 0.001, and *r*_*s*_ = 0.63, *n* = 33, *P* < 0.001, resp.).

Pain threshold was negatively correlated to impact on daily activities and quality of life (*r*_*s*_ = −0.44, *n* = 33, *P* < 0.001 and *r*_*s*_ = −0.43, *n* = 33, *P* < 0.001, resp.).

### 3.2. Impact of Systemic Inflammatory Activity on Daily Activities and Quality of Life

**Figure 2:**
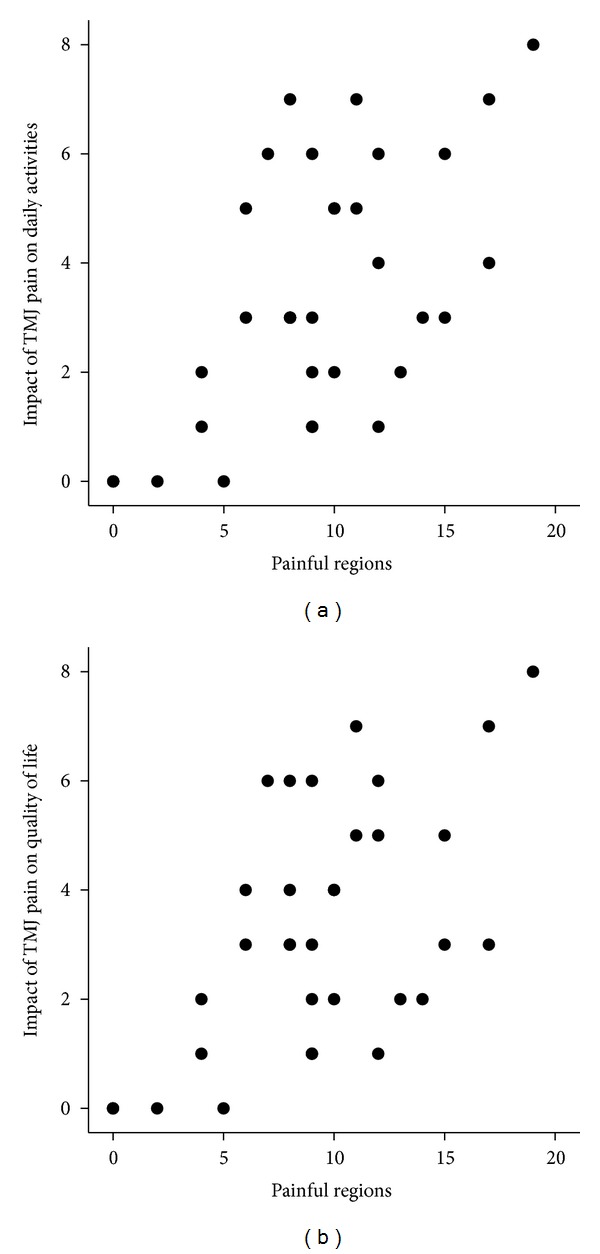
Scatter plots showing the relation between the number of painful regions and the impact of temporomandibular joint (TMJ) pain on daily activities ((a): *r*_*s*_ = 0.53, *n* = 33, *P* = 0.002) and quality of life ((b): *r*_*s*_ = 0.49, *n* = 33, *P* = 0.004).

DAS28 and number of painful regions were correlated to TMJ pain impact on daily activities (*r*_*s*_ = 0.43, *n* = 32, *P* = 0.014, and *r*_*s*_ = 0.53, *n* = 33, *P* = 0.002, resp.; Figure 2(a)) and quality of life (*r*_*s*_ = 0.37, *n* = 32, *P* = 0.038, and *r*_*s*_ = 0.49, *n* = 33, *P* = 0.004, resp.; Figure 2(b)).

DAS28 was in turn significantly related to number of painful regions (*r*_*s*_ = 0.67, *n* = 32, *P* < 0.001).

Partial correlation controlling for the influence of systemic inflammatory activity (DAS28) showed that number of painful movements and number of painful regions still had a significant impact, but to a lesser degree, on daily activities (*r*_*p*_ = 0.58, *n* = 29, *P* < 0.001 and *r*_*p*_ = 0.52, *n* = 29, *P* = 0.002, resp.) and quality of life (*r*_*p*_ = 0.55, *n* = 29, *P* < 0.001 and *r*_*p*_ = 0.54, *n* = 29, *P* = 0.002, resp.). Pain threshold was negatively related to both impact, on daily activities and quality of life (*r*_*p*_ = −0.39, *n* = 29, *P* = 0.029 and *r*_*p*_ = 0.37, *n* = 29, *P* = 0.041, resp.).

Partial correlation controlling for the influence of TMJ pain on movement (number of painful movements) showed that number of painful regions, one of the measures of systemic inflammatory activity, had a significant impact on daily activities (*r*_*p*_ = 0.43, *n* = 29, *P* = 0.015), and quality of life (*r*_*p*_ = 0.42, *n* = 29, *P* = 0.020).

### 3.3. Relation between TMJ Pain and Function

Maximum voluntary mouth opening capacity was negatively correlated to TMJ pain on chewing (*r*_*s*_ = −0.54, *n* = 33, *P* = 0.001).

TMJ pain threshold was negatively correlated with TMJ pain on chewing (*r*_*s*_ = −0.55, *n* = 33, *P* = 0.001), tenderness to palpation (*r*_*s*_ = −0.45, *n* = 33, *P* = 0.009), and TMJ pain on maximum mouth opening (*r*_*s*_ = −0.52, *n* = 33, *P* = 0.002) but was positively correlated to maximum mouth opening capacity (*r*_*s*_ = 0.72, *n* = 33, *P* < 0.001).

Anterior open bite was not significantly correlated to any other variable.

## 4. Discussion

This study indicates that both current TMJ pain intensity and systemic inflammatory activity are interactive factors behind the impact of TMJ pain on daily living and quality of life in RA. This implicates that TMJ treatment planning and prognosis estimation in RA patients should consider not only the TMJ pain intensity but also the systemic inflammatory activity.

The main finding of this study is that the systemic inflammatory activity is an important factor behind the degree of impact of TMJ pain. The study also confirmed the substantial impact of TMJ pain on daily activities and quality of life. Alstergren et al. showed that TMJ pain on mandibular movement was the strongest predictor of an inflammatory intra-articular milieu, that is, indicating an active local inflammation [11]. The fact that TMJ pain intensity on movement in this study was a strong predictor, together with number of painful regions, for the degree of impact of TMJ pain on daily activities and quality of life indicates that the inflammatory activity is an important determinant for the consequences of the disease. Indeed, the different TMJ pain entities (rest, movement, loading, palpation, etc.) have been found to be both locally and systemically modulated in RA but to various degrees, which support the findings in the present study [11]. This implicates that TMJ treatment planning and prognosis estimation in RA patients should consider not only the TMJ pain intensity but also the systemic inflammatory activity.

The disease RA impacts many aspects of daily life due to pain, stiffness, and functional restriction [12]. RA is commonly accompanied by local joint pain that increases during movements. Indeed, TMJ pain on movement and loading was related to the impact on daily activities and quality of life in this study. These findings are supported by previous studies where TMJ pain was found to have a substantial negative impact on activities of daily living in RA [1, 10, 13, 14]. Voog et al. [10] showed that the activities of daily living were negatively influenced in all included RA patients at different levels. The impact on daily living by TMJ pain/discomfort was greatest on the performance of physical exercises and jaw movements. Pain during maximum mouth opening was associated with difficulties with several activities such as to yawnly and opening the mouth widely.

In the arthritic synovial membrane, inflammatory mediators like cytokines are released from immunoactive cells to bind to local target receptors. To some extent, the cytokines “spill over” in to the blood and will eventually reach the liver. Cytokines like IL-1*β* and IL-6 in the circulation have been shown to activate afferent portions of the vagus nerve passing through the liver, causing or modulating the “illness response” when the nerve signals reach the vagus centers in the brain. The illness response comprises fever, malaise, mood changes, reduced appetite and libido, fatigue, increased or disturbed sleep as well as a disturbed central pain modulation [15, 16]. The illness response may therefore partly explain how TMJ inflammation may influence central pain processing, in addition to the peripheral sensitization of nociceptive nerve endings. Also, the cytokines found in blood increases the inflammatory markers of systemic inflammatory activity, that is, the TMJ inflammation influences the systemic inflammatory activity and central pain processing.

A combination of peripheral and systemic factors modulates TMJ pain in RA [11]. In the present study TMJ pain at rest, on movement and on loading (chewing) as well as palpatory tenderness was investigated. These variables represent pain entities with, to some extent, separate pain mechanisms where TMJ pain on movement and on chewing represents intra/periarticular mechanical sensitization, palpatory tenderness represents mechanical sensitization from external pressure, and pain at rest represents a mix of central and peripheral sensitization with endogenous activation of the nociceptive system. However, chronic pain may also be maintained by central mechanisms, for example, in the trigeminal centers in the brain stem and other brain centers for affective and cognitive aspects of pain, without a substantial sensory influx in the nociceptive system [17]. This may amplify all investigated pain aspects and also contribute to TMJ pain at rest. The greater impact of TMJ pain on patients with high systemic inflammatory activity as well as the finding that higher systemic inflammatory activity was associated with TMJ pain at mouth opening and chewing supports this.

A limitation of the study is that only one question regarding the impact on daily activities and quality of life, respectively, was used. There are validated instruments for assessment of activity of daily living or quality of life, including interference of disease aspects in these aspects. Such instruments should be considered in future studies. However, we consider that our questions are relevant and that the answers can be used as a broad measurement of the impact of TMJ pain as used in the present study.

### 4.1. Conclusion

In conclusion, this study indicates that both current TMJ pain intensity and systemic inflammatory activity play roles in the impact of TMJ pain on daily living and quality of life in RA. This implicates that TMJ treatment planning and prognosis estimation in RA patients should not only consider the TMJ pain intensity but also the systemic inflammatory activity.

## Figures and Tables

**Table 1:** Demographic and background data for 33 patients with temporomandibular joint (TMJ) involvement of rheumatoid arthritis.

		Median	Percentile		% abn	*n*
			25th	75th		
Age	Years	47	41	54		33
Gender	F/M					29/4
Duration						
General disease	Years	6	5	11		33
TMJ symptoms	Years	2	1	4		33
Smoking	Yes/no					5/28
Systemic disease activity						
Disease activity score (28)	0–10	3.9	3.5	4.9		32
Number of painful regions	0–21	9	8	12		33
Erythrocyte sedimentation rate	mm/hr	40	28	70	85	33
C-reactive protein	mg/L	8	6	11	53	26
Rheumatoid factor	IU/mL	27	25	34	84	26
ACPA					42	26
Thrombocyte particle count	10^9^/L	306	246	342	9	33
Medication						
NSAID					%	94
DMARD					%	100
Glucocorticoid					%	3
Anti-TNF					%	46

% abn: percentage of observations with positive or abnormal values (when applicable), *n*: number of observations, M: males, F: females, IU: international units, NSAID: nonsteroidal anti-inflammatory drug, DMARD: disease-modifying antirheumatic drug, anti-TNF: biologic drug specifically targeting tumor necrosis factor. The following values were considered abnormal: rheumatoid factor >14 IU, C-reactive protein >4 mg/L, erythrocyte sedimentation rate >20 mm/h, thrombocyte particle count >300 × 10^9^/L, and ACPA >5 U/mL. The disease activity score for 28 joints was assessed at the time of clinical examination.

**Table 2:** Clinical findings in 33 patients with temporomandibular joint (TMJ) involvement of rheumatoid arthritis.

		Median	Percentile		*n *
			25th	75th	
Impact of TMj pain					
On daily activity	NRS 0–10	3	2	5	33
Quality of life	NRS 0–10	3	2	5	33
TMJ pain intensity					
At rest	NRS 0–20	2	0	4	33
On maximum mouth opening	NRS 0–20	9	5	11	33
On chewing	NRS 0–20	4	2	8	33
Pain on movements	NRS 0–80	19	12	27	33
Mouth opening capacity					
Pain threshold	mm	35	28	40	33
Maximum mouth opening	mm	45	35	50	33
Tenderness to digital palpation	0–8	4	2	4	33
Number of teeth	0–32	29	27	31	33
Anterior open bite	0–9	4	2	7	33

NRS: numerical rating scale to assess the impact of TMJ pain on the patients abilities to perform daily activities and on the quality of their life on a 0–10 NRS where 0 corresponded to “no impact of TMJ pain on daily activities/quality of life” and 10 corresponded to “maximum impact of the TMJ pain on daily activities/quality of life.” A NRS was also used to assess the TMJ pain intensity where 0 corresponded to “no pain” and 10 to “worst imaginable pain.”
